# Supplementary material for: EnzML: multi-label prediction of enzyme classes using InterPro signatures
Source: BMC Bioinformatics. 2012 Apr 25;13:61. doi: 10.1186/1471-2105-13-61 (PMC3483700; doi:10.1186/1471-2105-13-61)
Supplement: Addtional file 5 — The Java code to format the data files, evaluate and predict. The file enzml_java_code.tar.gz contains the Java code used to format database data to ARFF and XML formats, to execute cross and train-test (jackknife) evaluations and to record evaluation results to database. More information is included in the readme.txt file and the Javadoc files. The code can be used with a MySQL database. To use a different database software, other JDBC drivers might be required. [file 1471-2105-13-61-S5.gz › java_code/enzml2011/doc/uk/ac/ed/inf/csb/taweka/DataSetFactoryTest.html]

DataSetFactoryTest


---


|  |  |  |
| --- | --- | --- |
| **Package** \| **Class** \| **Use** \| **Tree** \| **Deprecated** \| **Index** \| **Help** | |  |
| **PREV CLASS**   **NEXT CLASS** | **FRAMES**    **NO FRAMES**     **All Classes** |  |
| SUMMARY: NESTED \| FIELD \| CONSTR \| METHOD | DETAIL: FIELD \| CONSTR \| METHOD |  |


---


## uk.ac.ed.inf.csb.taweka Class DataSetFactoryTest

```
java.lang.Object
  junit.framework.Assert
      junit.framework.TestCase
          uk.ac.ed.inf.csb.taweka.DataSetFactoryTest
```

**All Implemented Interfaces:**: junit.framework.Test

---

``` public class DataSetFactoryTest extends junit.framework.TestCase ```

Class

**Version:**
:   20 Feb 2008

**Author:**
:   Luna De Ferrari luna.deferrari-at-ed.ac.uk

---

| **Field Summary** | |
| --- | --- |
| `static java.lang.String` | `arffInstance1` |
| `static java.lang.String` | `arffInstance2` |
| `static java.lang.String` | `arffInstance3` |
| `static java.lang.String` | `ATT1` |
| `static java.lang.String` | `ATT2` |
| `static java.lang.String` | `ATT3` |
| `static java.lang.String` | `CLASS1` |
| `static java.lang.String` | `CLASS2` |
| `static java.lang.String` | `INSTA` |
| `static java.lang.String` | `INSTB` |
| `static java.lang.String` | `INSTC` |
| `static java.lang.String` | `NAME` |


**Constructor Summary**

`DataSetFactoryTest()`


| **Method Summary** | |
| --- | --- |
| `static java.util.Vector<java.lang.String>` | `attributes()`             Data: ATT1 ATT2 ATT3 CLASS instA y n n 1 instB y y n 2 instC n n y 2 |
| `static java.util.Vector<java.lang.String>` | `classValues()` |
| `static DataSetFactory` | `dataSet()` |
| `static java.util.Vector<java.lang.String>` | `instances()` |
| `static void` | `main(java.lang.String[] args)`             Main method to test |
| `void` | `testAddDummyToClass2()`             test the method |
| `void` | `testCreateDataSet()` |
| `void` | `testGetArff()`             public void testInstances(){ DataSetFactory data = DataSetFactoryTest.dataSet(); Instances instances = data.getDataSet(); Enumeration instancesList = instances.enumerateInstances(); // instance 1 //Instance instance1 = (Instance) instancesList.nextElement(); //String instanceString1 = instance1.toString(); //assertEquals(DataSetFactoryTest.INSTA, instance1.name()); //assertEquals("{0 y,3 value1}", instanceString1); arffInstance1 = "{0 y,3 value1}"; arffInstance2 = "{0 y,1 y,3 value2}"; arffInstance3 = "{2 y,3 value2}"; } |
| `void` | `testGetAttributes()` |
| `void` | `testGetName()` |
| `void` | `testLog()` |
| `void` | `testSetClass()` |

**Methods inherited from class junit.framework.TestCase**

`countTestCases, getName, run, run, runBare, setName, toString`

**Methods inherited from class junit.framework.Assert**

`assertEquals, assertEquals, assertEquals, assertEquals, assertEquals, assertEquals, assertEquals, assertEquals, assertEquals, assertEquals, assertEquals, assertEquals, assertEquals, assertEquals, assertEquals, assertEquals, assertEquals, assertEquals, assertEquals, assertEquals, assertFalse, assertFalse, assertNotNull, assertNotNull, assertNotSame, assertNotSame, assertNull, assertNull, assertSame, assertSame, assertTrue, assertTrue, fail, fail, failNotEquals, failNotSame, failSame`

**Methods inherited from class java.lang.Object**

`equals, getClass, hashCode, notify, notifyAll, wait, wait, wait`

**Field Detail**

### NAME

```
public static final java.lang.String NAME
```

**See Also:**: Constant Field Values

---


### ATT1

```
public static final java.lang.String ATT1
```

**See Also:**: Constant Field Values

---


### ATT2

```
public static final java.lang.String ATT2
```

**See Also:**: Constant Field Values

---


### ATT3

```
public static final java.lang.String ATT3
```

**See Also:**: Constant Field Values

---


### INSTA

```
public static final java.lang.String INSTA
```

**See Also:**: Constant Field Values

---


### INSTB

```
public static final java.lang.String INSTB
```

**See Also:**: Constant Field Values

---


### INSTC

```
public static final java.lang.String INSTC
```

**See Also:**: Constant Field Values

---


### CLASS1

```
public static final java.lang.String CLASS1
```

**See Also:**: Constant Field Values

---


### CLASS2

```
public static final java.lang.String CLASS2
```

**See Also:**: Constant Field Values

---


### arffInstance1

```
public static final java.lang.String arffInstance1
```

**See Also:**: Constant Field Values

---


### arffInstance2

```
public static final java.lang.String arffInstance2
```

**See Also:**: Constant Field Values

---


### arffInstance3

```
public static final java.lang.String arffInstance3
```

**See Also:**: Constant Field Values


**Constructor Detail**

### DataSetFactoryTest

```
public DataSetFactoryTest()
```


**Method Detail**

### attributes

```
public static java.util.Vector<java.lang.String> attributes()
```

:   Data:
    ATT1 ATT2 ATT3 CLASS
    instA y n n 1
    instB y y n 2
    instC n n y 2

---


### instances

```
public static java.util.Vector<java.lang.String> instances()
```

---


### classValues

```
public static java.util.Vector<java.lang.String> classValues()
```

---


### dataSet

```
public static DataSetFactory dataSet()
```

---


### testSetClass

```
public void testSetClass()
```

---


### testGetName

```
public void testGetName()
```

---


### testGetAttributes

```
public void testGetAttributes()
```

---


### testCreateDataSet

```
public void testCreateDataSet()
```

---


### testLog

```
public void testLog()
```

---


### testGetArff

```
public void testGetArff()
```

:   public void testInstances(){
    DataSetFactory data = DataSetFactoryTest.dataSet();
    Instances instances = data.getDataSet();
    Enumeration instancesList = instances.enumerateInstances();
    // instance 1
    //Instance instance1 = (Instance) instancesList.nextElement();
    //String instanceString1 = instance1.toString();
    //assertEquals(DataSetFactoryTest.INSTA, instance1.name());
    //assertEquals("{0 y,3 value1}", instanceString1);
    arffInstance1 = "{0 y,3 value1}";
    arffInstance2 = "{0 y,1 y,3 value2}";
    arffInstance3 = "{2 y,3 value2}";
    }

---


### testAddDummyToClass2

```
public void testAddDummyToClass2()
```

:   test the method

---


### main

```
public static void main(java.lang.String[] args)
```

:   Main method to test

    :   **Parameters:**: `args` - should contain the name of an input file.


---


|  |  |  |
| --- | --- | --- |
| **Package** \| **Class** \| **Use** \| **Tree** \| **Deprecated** \| **Index** \| **Help** | |  |
| **PREV CLASS**   **NEXT CLASS** | **FRAMES**    **NO FRAMES**     **All Classes** |  |
| SUMMARY: NESTED \| FIELD \| CONSTR \| METHOD | DETAIL: FIELD \| CONSTR \| METHOD |  |


---
